# Extracellular Fragmented Self-DNA Is Involved in Plant Responses to Biotic Stress

**DOI:** 10.3389/fpls.2021.686121

**Published:** 2021-07-26

**Authors:** Francesca Barbero, Michela Guglielmotto, Monirul Islam, Massimo E. Maffei

**Affiliations:** ^1^Department of Life Sciences and Systems Biology, University of Turin, Turin, Italy; ^2^Neuroscience Institute of Cavalieri Ottolenghi Foundation, University of Turin, Turin, Italy; ^3^Plant Physiology Unit, Department of Life Sciences and Systems Biology, University of Turin, Turin, Italy

**Keywords:** tomato, transmembrane potential, calcium signaling, ROS, ion channel activity, RNA-seq, receptor-like kinase, ethylene-responsive elements

## Abstract

A growing body of evidence indicates that extracellular fragmented self-DNA (eDNA), by acting as a signaling molecule, triggers inhibitory effects on conspecific plants and functions as a damage-associated molecular pattern (DAMP). To evaluate early and late events in DAMP-dependent responses to eDNA, we extracted, fragmented, and applied the tomato (*Solanum lycopersicum*) eDNA to tomato leaves. Non-sonicated, intact self-DNA (intact DNA) was used as control. Early event analyses included the evaluation of plasma transmembrane potentials (Vm), cytosolic calcium variations (Ca^2+^_cy__t_), the activity and subcellular localization of both voltage-gated and ligand-gated rectified K^+^ channels, and the reactive oxygen species (ROS) subcellular localization and quantification. Late events included RNA-Seq transcriptomic analysis and qPCR validation of gene expression of tomato leaves exposed to tomato eDNA. Application of eDNA induced a concentration-dependent Vm depolarization which was correlated to an increase in (Ca^2+^)_cyt_; this event was associated to the opening of K^+^ channels, with particular action on ligand-gated rectified K^+^ channels. Both eDNA-dependent (Ca^2+^)_cyt_ and K^+^ increases were correlated to ROS production. In contrast, application of intact DNA produced no effects. The plant response to eDNA was the modulation of the expression of genes involved in plant–biotic interactions including pathogenesis-related proteins (PRPs), calcium-dependent protein kinases (CPK1), heat shock transcription factors (Hsf), heat shock proteins (Hsp), receptor-like kinases (RLKs), and ethylene-responsive factors (ERFs). Several genes involved in calcium signaling, ROS scavenging and ion homeostasis were also modulated by application of eDNA. Shared elements among the transcriptional response to eDNA and to biotic stress indicate that eDNA might be a common DAMP that triggers plant responses to pathogens and herbivores, particularly to those that intensive plant cell disruption or cell death. Our results suggest the intriguing hypothesis that some of the plant reactions to pathogens and herbivores might be due to DNA degradation, especially when associated to the plant cell disruption. Fragmented DNA would then become an important and powerful elicitor able to trigger early and late responses to biotic stress.

## Introduction

Although it is not fully understood how it is generated, extracellular fragmented DNA (eDNA) contributes to the species-specific discrimination of self- versus non-self (Duran-Flores and Heil, 2018) and can be used by plants to build resistance against the surrounding environment (Gallucci and Maffei, 2017). One of the most stimulating perspectives in plant crop production is the application of self-eDNA to drive responses similar to the intrinsic DNA damage response. A growing body of evidence indicates that extracellular fragmented self-DNA, by acting as a signaling molecule, might be able to trigger inhibitory effects on conspecific plants and function as a damage-associated molecular pattern (DAMP) (Mazzoleni et al., 2015). To explain eDNA action, two general mechanisms have been proposed: the presence of membrane receptors able to trigger a signal transduction cascade of events or the possibility that fragmented DNA may enter somehow into the cytosol and interfere with some biological processes (Duran-Flores and Heil, 2015). In support of the first hypothesis are results showing that eDNA can trigger very early events, like the membrane depolarization and the cytosolic influx of calcium ions in a dose-dependent manner (Barbero et al., 2016). Moreover, the persistence of a membrane depolarization after the washing out of eDNA suggested that eDNA might interact with either membrane receptors or ligand-gated ion channels (Barbero et al., 2016). However, to date, no plant receptors able to recognize fragments of eDNA with a level of sequence-specificity have been reported.

An open question remains whether eDNA acts directly on the plant cell and provokes growth-inhibition effects (Mazzoleni et al., 2015) or acts as a DAMP and plays a role as an elicitor. In plant roots, DNA is excreted and released to the root cap environment by lytic processes (Driouich et al., 2019), but DNA could also be degraded by infection and disruption of root cap cells (Monticolo et al., 2020). In the latter case, eDNA might be involved in plant responses to biotic stress and could be released along with other elicitors in the extracellular environment (Plancot et al., 2013). Interesting, pathogen’s eDNA also plays a role, as recently reported by Serrano-Jamaica et al. (2021), who found that the foliar application of eDNA from the pathogens *Phytophthora capsici*, *Fusarium oxysporum*, and *Rhizoctonia solani* triggers plant defense pathways. Therefore, it appears that both plant and pathogen eDNAs can prompt plant responses both above and belowground.

Although with different strategies and rates, by feeding on plants, some herbivore and pathogens disrupt cell integrity and generate the leakage of ions, the delivery of lytic enzymes from lysosomes, and the degradation of organelles and their content. A common reaction to this devastating event is the alteration of the plasma transmembrane potential (Vm), the production of reactive oxygen species (ROS), and the triggering of calcium signaling, that eventually leads to local and systemic modulation of biotic stress-responding genes (Maffei et al., 2007; Bricchi et al., 2012, 2013; Zebelo and Maffei, 2015). Several elicitors of plant responses to biotic stress have been characterized (Maffei et al., 2012) and specific receptors have been described (Iida et al., 2019); however, since most of the biotic stress causes cell disruption, DNA degradation, and fragmentation cannot be excluded and eDNA might interfere with elicitors and receptors.

The current knowledge on eDNA effects on plants still lacks the demonstration that application of self-eDNA to a plant may trigger both early events (mostly involving the plant cell plasma membrane) and the signal transduction pathway that leads to gene expression. Therefore, the aim of this work is to assess whether plant eDNA can elicit specific plant reactions as found in response to biotic stress. In order to verify this hypothesis, we extracted and fragmented tomato (*Solanum lycopersicum*) DNA and tested its effects on tomato leaves by evaluating early (Vm, calcium, and potassium channel activity, ROS generation) and late events (gene expression by RNA-Seq and qPCR analyses). Here we show that application of eDNA can induce tomato early and late events, with pattern similar to those described for plant responses to biotic stress.

## Materials and Methods

### Plant Material and Sampling

Tomato *S. lycopersicum* L. seeds cv “cuore di bue” (Franchi seed company, Italy) were sown in glass plates with wet filter paper and incubated in a growth chamber (25°C, 16/8 h light/dark, PPFD 100 μmol m^–2^ s^–1^) for 5 days. Seedlings were then transferred in polyethylene plastic pots (8 cm ∅) containing a mixture of peat, soil (Klasmann-Deilmann, Germany), sand, and vermiculite (Unistara, Italy) and grown in plant growth chambers with a light intensity of 120 μmol m^–2^ s^–1^. Plants were watered three times a week and fertilized twice a week with a 0.1% solution containing N:P:K (12:10:10). Experiments were conducted with 20- to 30-day-old seedlings by sampling expanded leaves.

For all studies, we used mechanical damage in order to allow eDNA to penetrate the leaf tissues, as reported earlier (Barbero et al., 2016). In particular, for Vm analyses, plant responses were induced on mechanically damaged tomato leaves by tomato eDNA. A pattern wheel was used to simulate a mechanical damage for all microscopic studies. As negative controls, undamaged leaves were used, and in order to compare the effect of eDNA with controls, we defined the timing of wounding at 30 min. That is, the application was performed continuously for 30 min, while mechanical damage was performed once. For molecular studies, plants were exposed to eDNA for 1 h, and then the total RNA was extracted from control and treatments.

### DNA Extraction and Sonication

Leaves of tomato were collected and dried in oven at 60°C for 72 h. For DNA extraction, 800 mg of dried material were ground to powder in liquid nitrogen with mortar and pestle. Total DNA was isolated using both cetyl trimethyl ammonium bromide (CTAB) method, according to Wilke’s protocol (Wilke, 1997) and a DNeasy Plant Mini Kit as described by the manufacturer (Qiagen, Valencia, CA, United States^1^). Briefly, PVPP (Polyvinylpolypyrrolidone, Sigma, Milan) powder was added to the tissue before grinding. Tissues were homogenized with 10 ml of extraction buffer (100 mM Tris–HCl, pH 8.0, 1.4 M NaCl, 0.02 mM EDTA, 2% CTAB, and 0.2% β-mercaptoethanol). After centrifugation (13,000 rpm for 10 min), an equal volume of chloroform:isoamyl alcohol (24:1) was added, and samples were centrifuged again (13,000 rpm for 20 min). We repeated this step and, after incubation for 30 min with a 1:100 volume of RNAse, the DNA was precipitated with isopropanol. Then samples were centrifuged at 13,000 rpm for 10 min, and the DNA pellet was washed twice with 76% aqueous ethanol, 0.2 M sodium acetate, and 70% aqueous ethanol subsequently. Finally, the pellet was air-dried and resuspended in PE buffer (5 mM Tris/HCl, pH 8.5).

DNA from tomato leaves was fragmented by sonication using a Bandelin Sonopulse HD2070 (Bandelin, Berlin, Germany) at 90% power with a 1 s pulse for 15 min. Following the manufacturer’s recommendation, samples were maintained in a layer of ice during the sonication process (see Barbero et al., 2016 for further details on the protocol). Capillary gel electrophoresis with the Agilent 2100 Bioanalyzer (Agilent Technologies) was used to assess quality and length of sonicated band sizes, according to manufacturer’s instructions. DNA extract was spectrophotometrically quantified at 260 nm on a NanoDrop ND 1000 Spectrophotometer (Thermo Fisher Scientific, Wilmington, DE, United States) and visually verified on 1.2% agarose gel using Gel Doc EZ System (Bio-rad, CA, United States).

### Determination of Transmembrane Potentials

The transmembrane potential (Vm) was determined in leaf segments with glass micropipettes with a tip resistance of 4–10 MΩ and filled with 3 M KCl as previously detailed (Maffei and Bossi, 2006). Leaf segments were settled for 60–120 min in 5 mM Mes-NaOH (pH 6.0). Perfusion of the buffer was obtained by a multichannel Ismatec Reglo (Ismatec SA, Glattbrugg, Switzerland) peristaltic pump (flow rate 1 ml min^–1^). Based on the topographical and temporal determination of Vm performed previously, the electrode was inserted between 0.5 and 1.5 mm from the leaf edge zone. Vm variations were recorded through a PC digital port with a data logger. eDNA was assayed at 50 and 100 μg ml^–1^. 0.05 M KCl was used as a control of Vm depolarization, according to Barbero et al. (2016).

### Evaluations of Intracellular Calcium Variations by Confocal Laser Scanning Microscopy (CLSM) and Calcium Orange

Calcium Orange dye (stock solution in DMSO, Molecular Probes, Leiden, Netherlands) was diluted in 5 mM MES-Na buffer (pH 6.0) to a final concentration of 5 μM. This solution was applied to tomato leaves attached to the plant, as previously reported (Maffei et al., 2004; Bricchi et al., 2010; Barbero et al., 2016). After 1 h incubation with Calcium Orange, the leaf was mounted on a Leica TCS SP2 (Leica Microsystems Srl, Milan, Italy) multiband confocal laser scanning microscopy stage without separating the leaf from the plant in order to assess the basic fluorescence levels as a control. Then 50 μl of 200 μg ml^–1^ of eDNA was applied and after 30 min the calcium signature was observed. The microscope operates with a Krypton/Argon laser at 543 and 568 nm wavelengths: the first wavelength excites Calcium Orange, resulting in green fluorescence and the second mainly excites chlorophyll, resulting in red fluorescence. All images were obtained with an objective HCX APO 40× in water immersion with an NA of 0.8. Scan speed was set at 400. The microscope pinhole was 0.064 mm and the average size depth of images was between 65 and 70 μm; the average number of section per image was 25 and the pictures were represented as the merging of stacks. Image format was 1024 × 1024 pixels, 8 bits per sample and 1 sample per pixel. At least five plants were used for each experiment and the CLSM analyses were performed on different leaves.

### CLSM Localization of Voltage- and Ligand-Gated K^+^ Channels Using FluxOR^*TM*^

Voltage-gated K^+^ channels were assayed by using the FluxOR^*TM*^ potassium ion channel kit from Invitrogen (Molecular Probes). Non-detached tomato leaves were gently placed on a glass slide and incubated in the dark for 1 h with 100 μl of loading buffer (deionized water, FluxOR^*TM*^ assay buffer, and probenecid) by following the manufacturer’s instructions. Plants were treated with 50 μl of 200 μg ml^–1^ of eDNA as above. Just before observation 50 μl of stimulus buffer (deionized water, FluxOR^*TM*^ chloride-free buffer, K_2_SO_4_, and Tl_2_SO_4_) were added by following the manufacturer’s instructions. CLSM fluorescence was assayed by a Leica TCS SP2 microscope equipped with an argon laser (excitation wavelength of 488 nm). Fluorescence was visible after about 50 min from treatment. Emissions were recorded using a 520–535 nm bandpass filter as detailed previously (Bricchi et al., 2013).

### CLSM Subcellular Localization of H_2_O_2_ and Active Peroxidases by Using 10-Acetyl-3,7-Dihydroxyphenoxazine (Amplex Red)

Tomato leaves from rooted plants in pot were treated with 50 μl of 200 μg ml^–1^ of eDNA after incubation with the dye 10-acetyl-3,7-dihydroxyphenoxazine (Amplex Red) as described earlier (Maffei et al., 2006). The Molecular Probes Amplex Red Hydrogen Peroxide/Peroxidase Assay kit (A-22188) was used. The Assay Kit contains a sensitive, one-step assay that uses the Amplex^®^ Red reagent (10-acetyl-3,7-dihydroxyphenoxazine) to detect hydrogen peroxide (H_2_O_2_) or peroxidase activity. The Amplex^®^ Red reagent, in combination with horseradish peroxidase (HRP), was used to detect H_2_O_2_ released from eDNA treated leaves or generated in enzyme-coupled reactions after eDNA application. The reagent was dissolved in MES-Na buffer 50 mM (pH 6.0) containing 0.5 mM calcium sulfate to obtain a 50 μM solution. Leaves were then mounted on a Leica TCS SP2 microscope as described above. Scannings were recorded after 180 min using the HCX PL APO 63×/1.20 W Corr/0.17CS objective. The microscope was operated with a Laser Ar (458 nm/5 mW; 476 nm/5 mW; 488 nm/20 mW; 514 nm/20 mW), a Laser HeNe 543 nm/1.20 mW, and a Laser HeNe 633 nm/10 mW.

### RNA-Seq Data Processing and Analysis

To assess the effect of eDNA on tomato gene expression, four plants were treated with 200 ng μl^–1^ eDNA as described above. Four controls were represented by intact DNA. After 1 h treatment, leaves were immediately harvested in liquid nitrogen and stored at −80°C for subsequent analysis.

Total RNA was isolated using TRIzol kit (Invitrogen), according to the manufacturer’s protocol. Quantity and quality of the starting RNA were checked by Qubit and Bioanalyzer (Agilent), and libraries were prepared using the TruSeq RNA Sample Prep Kit (Illumina), following the manufacturer’s instructions. Sequencing was performed on the Illumina NextSeq 500 platform. After quality controls with FastQC,^2^ sequencing reads were aligned to *S. lycopersicum* (tomato) 2.50 genome reference (SL2.50) using TopHat v2.0.13 (Kim et al., 2013). Gene expression levels were quantified with the “HTSeq” framework v0.6.1 (Anders et al., 2015), using the International Tomato Genome Sequencing Project (ITAG) v2.4 gene/transcripts annotation. Differential expression analysis was carried out with the DESeq2 (Love et al., 2014) R/Bioconductor package.

### Validation of RNA-Seq Gene Expression by qRT-PCR

Samples as above were used for the qPCR analyses which were run on a QUANTSTUDIO 3 Real-Time System (Thermo Fisher Scientific, Waltham, MA, United States) using SYBR green I with ROX as an internal loading standard. The reaction mixture was 10 μL, comprising 5 μL of 2X Maxima^*TM*^ SYBR Green qPCR Master Mix (Maxima SYBR Green/ROX qPCR Master Mix 2X, Thermo Fisher Scientific, United States), 0.5 μL of 1:10 diluted cDNA and 100 nM primers (Integrated DNA Technologies, Coralville, IA, United States). Furthermore, non-templates were run as a negative control using only total RNA without reverse transcription to monitor for genomic DNA contamination and the same was done by using water with water. Primers were designed using Primer 3.0 software (Rozen and Skaletsky, 2000) as reported in Supplementary Table 1. The thermal conditions for all genes were: 10 min at 95°C, 40 cycles 15 s at 57°C, and 20 s at 72°C. Fluorescence was read following each annealing and extension phase. All runs were followed by a melting curve analysis from 55 to 95°C. The linear range of template concentration to threshold cycle value (Ct value) was determined by preparing a dilution series, using cDNA from three independent RNA extractions analyzed in three technical replicates. Primer efficiencies for all primer pairs were calculated using the standard curve method. All amplification plots were analyzed with the QUANTSTUDIO 3 software to obtain Ct values (Pfaffl, 2001).

The following groups of genes were analyzed: Calcium-related genes: calcium-binding EF hand family protein (*Solyc10g006700*), calmodulin (*Solyc04g058160*), calcium-binding phospholipase D (*Solyc01g091910*). Oxidative stress-related genes: ubiquinol oxidase (*Solyc08g075550*), polyphenol oxidase F, chloroplastic, PPO (*Solyc08g074630*), peroxidase (*Solyc03g025380*), catalase (*Solyc01g100630*). Proton pump-related genes: V-type proton ATPase subunit a (*Solyc11g072530*), proton pump interactor 1 (*Solyc08g068850*), proton pump interactor 1 (*Solyc05g008780*). Defense-related genes: 4-coumarate-CoA ligase-like protein (*Solyc06g035960*), β-1,3-glucanase (*Solyc01g060020*), chymotrypsin inhibitor-2 (*Solyc09g084450*), Kunitz-type protease inhibitor (*Solyc03g098780*), multidrug resistance protein ABC transporter family (*Solyc05g014500*), pathogenesis-related protein 1a (*Solyc01g106620*), pathogenesis-related protein P2 (*Solyc01g097240*), pathogenesis-related protein-1 (*Solyc01g106610*), polygalacturonase (*Solyc12g096730*), sesquiterpene synthase (*Solyc07g052130*), trypsin inhibitor-like protein precursor (*Solyc11g022590*), wound induced protein (*Solyc07g054780*), wound/stress protein lipoxygenase, LH2 PLAT domain-containing protein (*Solyc03g096550*), wound-induced proteinase inhibitor 1 (*Solyc09g084470*). Heat shock proteins (Hsps) and chaperones: heat shock protein Hsp90 (*Solyc07g047790*), heat shock protein (*Solyc03g117630*), heat shock protein 22 Mitochondrial (*Solyc08g078700*), heat shock protein 70 (*Solyc03g082920*), heat shock transcription factor 1 (*Solyc02g079180*), hsc1 heat shock protein 70 kDa (*Solyc06g076020*), hsc70.3 er21 ethylene-responsive heat shock protein cognate 70 (*Solyc04g011440*), hsp40 Chaperone protein (*Solyc11g071830*), hsp90 heat shock protein 90 (*Solyc06g036290*), NEF Heat shock protein 4 (*Solyc07g043560*), SHsfA7 Heat stress transcription factor A3 (*Solyc09g065660*). DNA binding: DNA primase/helicase (*Solyc02g022830*), DNA-directed RNA polymerase (*Solyc02g083350*). Receptor-like genes: receptor-like serine/threonine-protein kinase (*Solyc03g025130*), serine/threonine-protein kinase (*Solyc03g112950*), TIR-NBS-LRR resistance protein Toll-Interleukin receptor (*Solyc00g294230*), TIR-NBS-LRR disease resistance-like protein (*Solyc07g052790*), CC-NBS-LRR, resistance protein (*Solyc10g047320*). Phytohormone-related genes: ACC oxidase (*Solyc09g008560*), auxin-induced SAUR-like protein (*Solyc01g111000*), ethylene-responsive nuclear protein (Solyc02g070040), ethylene-responsive transcription factor 4 (*Solyc12g009240*), ethylene-responsive TF1 pathogenesis-related transcriptional factor (*Solyc03g093550*). Photosynthesis: chloroplastic RuBisCO small subunit 3B (*Solyc02g085950*), RuBisCO activase 1 (*Solyc09g011080*).

Four different reference genes TC194780a, actin 1 (*ACT1*); X14449, elongation factor 1α (*EF1*); DQ205342, β-tubulin (*TUB*), and TC193502a, ubiquitin (*UBI*) (see Supplementary Table 1 for primers), according to Løvdal and Lillo (2009). The best of the four genes was selected using the NormFinder software (Andersen et al., 2004). The relative expression mRNA levels of each gene were calibrated and normalized with the level of the most stable reference genes, *EF1* and *UBI* [in agreement with (Løvdal and Lillo, 2009)]. For each treatment, three biological replicates and three technical replicates were analyzed.

### Statistical Analyses

A stem-and-leaf function of SYSTAT 10 was used to treat Vm data to extract the lower and upper hinge from the Gaussian distribution. After filtering the data, the mean value was calculated along with the SE. At least five samples per treatment group were used for the statistical analysis of all other experimental data. Overall variations in the abundance of Calcium were assessed on Log-transformed data using the analysis of variance (ANOVA), while Tukey’s *post hoc* test was used to account for pairwise differences. Data are expressed as mean values ± SE.

## Results

### Tomato eDNA Induces a Transmembrane Potential Depolarization in Tomato Leaves

Sonication of DNA yielded fragments of different bp, in line with previous studies (Barbero et al., 2016). In particular, tomato eDNA consisted of fragments in the range between 250 and 1000 bp (data not shown). This eDNA was then used as a treatment.

**FIGURE 1:**
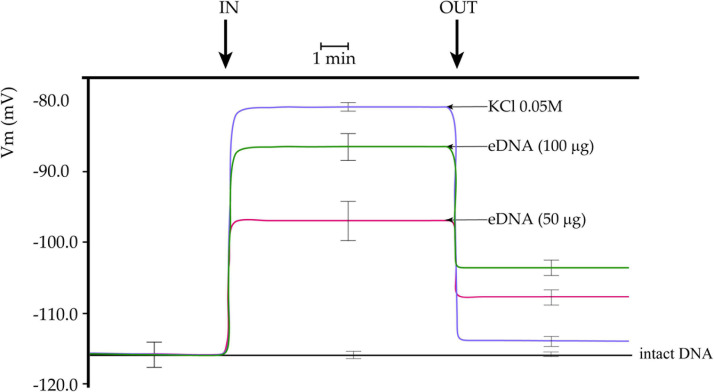
Plasma membrane potential (Vm) depolarization in response to different concentrations of tomato fragmented self-DNA (eDNA) on tomato leaves. No effect on Vm was found after non-fragmented self-DNA (intact DNA) application. 0.05 M KCl caused an expected and almost completely reversible Vm depolarization. Vm depolarization was dependent on eDNA concentration. Washing eDNA treated leaves with fresh buffer was unable to recover the tomato leaf Vm completely. A time scale is indicated. Error bars represent SE (*n* = 8–10). IN, timepoint when tomato cells were perfused with either KCl, eDNA, or intact DNA; OUT, timepoint when fresh buffer was perfused to wash out molecules.

We first assessed the effect of the application of tomato eDNA on tomato mesophyll cells by measuring variations in the leaf plasma transmembrane potential (Vm). We found that tomato leaves have an average mesophyll cell Vm ranging between 113 and 118 mV. Upon perfusion with non-fragmented tomato self-DNA (intact DNA), tomato Vm did not show any significant changes (Figure 1). To evaluate the tomato cell response to a Vm depolarizing agent, we perfused tomato leaves with a 0.05 M KCl solution. The effect was a sudden and constant Vm depolarization, which lowered the Vm at about 80 mV (Figure 1), as expected (Wakeel, 2013). We then perfused tomato cells with increasing concentrations of eDNA. The effect was a Vm depolarization directly correlated to the eDNA concentration, with 50 μg eDNA producing a 17 mV depolarization and 100 μg eDNA producing a 28 mV Vm depolarization, with respect to intact eDNA (Figure 1). While washing leaves with a fresh buffer returned the Vm value of KCl-induced Vm depolarization to almost initial values, the removal of the eDNA at both concentrations caused a Vm hyperpolarization that never reached the initial values. eDNA backwashed treatments at 50 μg eDNA and 100 μg eDNA reached a value of about −108 and −103 mV, respectively (Figure 1).

### Application of Tomato eDNA Is Associated With Increased Tomato Cytosolic Calcium Concentration

**FIGURE 2:**
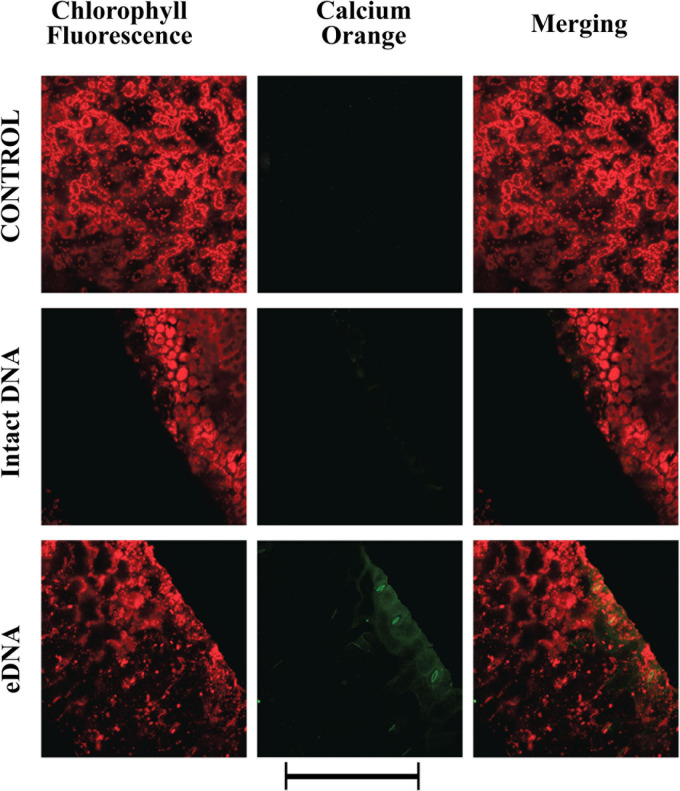
Intracellular calcium variations in tomato leaves in controls (no treatment) and upon treatments with tomato intact DNA and eDNA. False-color image analysis reconstructions from confocal laser-scanning microscope observations, and fluorochemical intracellular Ca^2+^ localization. Fifty microliters of 200 μg ml^–1^ of either eDNA or intact DNA were applied and after 30 min a calcium signature was observed. Pictures represent portions of the tomato leaf blade where the green fluorescence refers to the binding of calcium orange with Ca^2+^, whereas the chloroplasts are evidenced by a bright red color caused by chlorophyll fluorescence. Scale bar (250 μm) is indicated at the bottom of the figure. Pictures are the results of the merging of 25 individual optical sections.

Membrane depolarization depends on the differential distribution of ions across the plasma membrane (Maffei et al., 2007). To assess the response of tomato plants to self-eDNA on tomato cytosolic calcium (Ca^2+^_cyt_), we analyzed both localization and semi-quantitative evaluation of Ca^2+^_cyt_ by CLSM, using Calcium Orange as a selective calcium indicator (Kanchiswamy et al., 2014). A preliminary dose-dependent analysis allowed to assess that 200 μg ml^–1^ eDNA could induce a significant response (data not shown). Figure 2 shows the chlorophyll and calcium orange fluorescence as well as the merging of the two signals in controls (where no treatment is applied) and in leaves treated with 200 μg ml^–1^ of either intact DNA or eDNA. The images clearly show that only after eDNA application a strong fluorescence is associated with the Ca^2+^_cyt_ signature, whereas the signal observed after intact DNA had a similar fluorescence as the Calcium Orange control (Figure 2).

### Tomato Ligand-Gated and Voltage-Gated Potassium Channels Are Activated by the Application of Tomato eDNA

**FIGURE 3:**
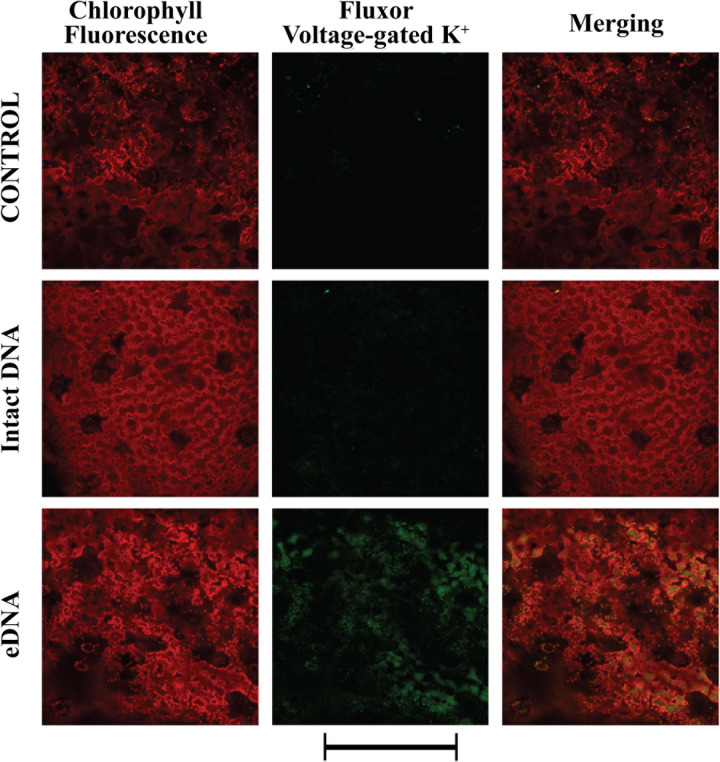
Intracellular potassium voltage-gated variations in tomato leaves in controls (no treatment) and upon treatments with tomato intact DNA and eDNA. False-color image analysis reconstructions from confocal laser-scanning microscope observations and fluorochemical evidence of voltage-gated activity. Fifty microliters of 200 μg ml^–1^ of either eDNA or intact DNA were applied and after 50 min the K^+^ signature was observed. Pictures represent portions of the tomato leaf blade where the green fluorescence refers to FluxOR^*TM*^ associated to voltage-gated K^+^ channel activity, whereas the chloroplasts are evidenced by a bright red color caused by chlorophyll fluorescence. Scale bar (250 μm) is indicated at the bottom of the figure. Pictures are the results of the merging of 25 individual optical sections.

To gain more insight into the possible causes of Vm depolarization, we tested the activity of both voltage- and ligand-gated K^+^ channels using the potassium indicator FluxOR^*TM*^. The assay is based on the use of a stimulus buffer containing a low level of thallium ions (Wible et al., 2008). Thallium ions freely flow through open K^+^ channels, acting as a surrogate for K^+^. When the K^+^ channel is stimulated by the presence of tomato eDNA, thallium flows into the cell and binds the FluxOR^*TM*^ dye, generating a fluorescent signal, proportional to channel activity. The fluorescent indicator measures ion flux in both voltage- and ligand-gated potassium channels. Voltage gated potassium channels are opened by the co-administration of potassium and thallium in the stimulus buffer. Resting and inward rectifier potassium channels are assayed by adding stimulus buffer with thallium alone. Figure 3 shows the localization of voltage-gated K^+^ channel activity. These channels are activated by variations in the Vm potential. We found that these channels’ activity followed in time the calcium signature and could be classified as inwardly rectifying (allowing K^+^ influx to the cell) (Cherel, 2004). A strong activity of these channels was found only after treatment with eDNA, whereas the intact DNA showed a signature similar to the control (Figure 3).

**FIGURE 4:**
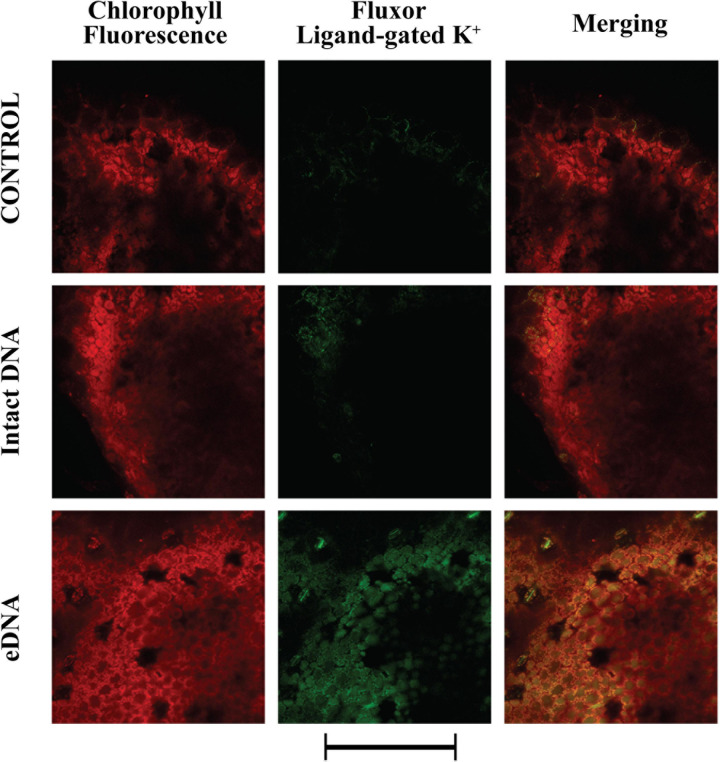
Intracellular potassium ligand-gated variations in tomato leaves in controls (no treatment) and upon treatments with tomato intact DNA and eDNA. False-color image analysis reconstructions from confocal laser-scanning microscope observations, and fluorochemical evidence of ligand-gated K^+^ channel activity. Fifty microliters of 200 μg ml^–1^ of either eDNA or intact DNA were applied and after 50 min the K^+^ signature was observed. Pictures represent portions of the tomato leaf blade where the green fluorescence refers to FluxOR^*TM*^ associated to ligand-gated K^+^ channel activity, whereas the chloroplasts are evidenced by a bright red color caused by chlorophyll fluorescence. Scale bar (250 μm) is indicated at the bottom of the figure. Pictures are the results of the merging of 25 individual optical sections.

We also evaluated the localization and activity of ligand-gated K^+^ channels. A strong and diffuse fluorescence was detected after treatment with eDNA (Figure 4). However, a low level of activity was also detected in both controls and treatment with intact DNA (Figure 4).

### Tomato Responses to eDNA Are Associated to the Generation of ROS

**FIGURE 5:**
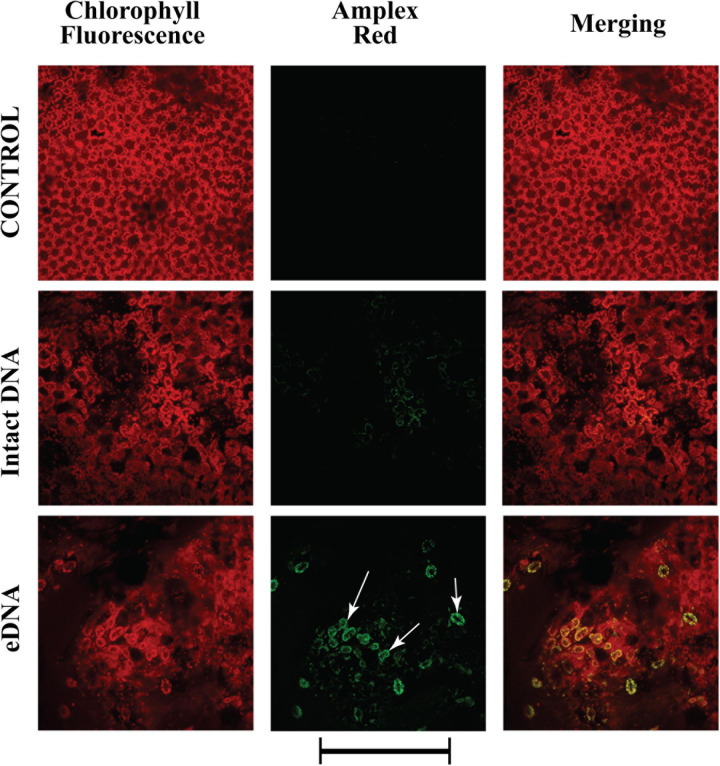
Intracellular variations of H_2_O_2_ production in tomato leaves in controls (no treatment) and upon treatments with tomato intact DNA and eDNA. The Amplex^®^ Red reagent, in combination with horseradish peroxidase (HRP), was used to detect H_2_O_2_ released or generated in enzyme-coupled reactions. False-color image analysis reconstructions from confocal laser-scanning microscope observations, fluorochemical H_2_O_2_, and peroxidase localization. Fifty microliters of 200 μg ml^–1^ of either eDNA or intact DNA were applied and after 180 min the ROS signature was observed in close association with chloroplasts (arrows). Pictures represent portions of the tomato leaf blade where the green fluorescence refers to the binding of Amplex^®^ Red with peroxidase-produced H_2_O_2_, whereas the chloroplasts are evidenced by a bright red color caused by chlorophyll fluorescence. Scale bar (250 μm) is indicated at the bottom of the figure. Pictures are the results of the merging of 25 individual optical sections.

Having assessed that the Vm depolarization is associated to a K^+^ influx and an influx of calcium in the cytosol, we assessed one of the characteristic responses following these early events: the activity of peroxidases and the production of a typical ROS, hydrogen peroxide (H_2_O_2_) (Zebelo and Maffei, 2015; Camejo et al., 2016). The Amplex^®^ Red reagent, combined with HRP, was used to detect H_2_O_2_ released from tomato leaves upon treatment with tomato eDNA. Application of intact DNA prompted a faint fluorescence reaction indicating the activity of peroxidases and the production of H_2_O_2_ (Figure 5). However, a stronger fluorescence, which appeared to be associated mostly with chloroplasts, was observed after applying tomato eDNA (Figure 5). Amplex^®^ Red reagent was also used as an ultrasensitive assay for peroxidase activity by using H_2_O_2_ in excess (data not shown), and the results were the same as with the use of HRP.

### The Tomato Response to eDNA Is Associated to the Modulation of Gene Expression

To assess the tomato responses to eDNA, we performed a transcriptomic analysis by RNA-Seq of fully expanded tomato leaves from 25 day-old plants grown in pots and treated with 200 μg ml^–1^ eDNA. Controls were represented by plants growing in the same conditions (i.e., temperature, gravity, atmospheric pressure, and Photosynthetic Flux Density) and treated with 200 μg ml^–1^ intact DNA. For significant analysis, genes were filtered based on their adjusted (FDR corrected) *P*-values calculated from the bioinformatic analysis. In general, almost all biological replicates analyzed were retained in the analysis and the genes satisfying a corrected *P*-value cut-off of 0.05 and fold change ≥ 2 ranged from 2 to 3% out of the total gene number (Supplementary Table 2). A total of 34,725 reads provided 845 DEGs of which 574 were downregulated and 271 upregulated. Several classes of genes were modulated including receptors, among them the TIR-NBS-LRR Toll-Interleukin receptor (*Solyc00g294230*).

We then analyzed the gene ontology (GO) of the biological processes of downregulated and upregulated genes and calculated the fold enrichment (FE) (i.e., the ratio between input data – i.e., the number of down/upregulated genes – versus the number of expected genes for each GO category, see Supplementary Table 3). Most of the downregulated genes (90%) showed a FE > 2, with particular reference among others to myo-inositol biosynthetic and metabolic processes (GO:0010264 and GO:0033517; >100 FE), nitric oxide biosynthetic and metabolic processes (GO:0006809 and GO:0046209; >70 FE), ROS biosynthetic process (GO:1903409; >70 FE), cell wall biosynthetic processes (GO:0031506 and GO:0000032; >47 FE), jasmonic acid biosynthetic process (GO:0009695; >47 FE), and sucrose transport (GO:0015770; >47 FE) (Supplementary Figure 1).

Many upregulated genes (47%) showed a GO FE > 2. Among these, oxygen transport (GO:0015671; >66 FE), defense response to Gram-negative bacterium (GO:0050829; >66 FE), lactate biosynthetic process (GO:0019249; >66 FE), adenine biosynthetic, metabolic, and salvage processes (GO:0046084, GO:0046083, GO:0006168; >33 FE), auxin influx (GO:0060919; >33 FE), and several cellular ion homeostasis processes (GO:0030002, GO:0072501, GO:0030320, GO:0030643, GO:0072502, GO:0072505, GO:0055083, GO:0055062, GO:0072506; all > 33 FE as well as GO:0046916, GO:0055076, GO:0006875, GO:0055065, GO:0055082, GO:0019725, GO:0098771, GO:0030003, GO:0050801, GO:0030001; all > 2 FE) (Supplementary Figure 2).

We then processed the downregulated and upregulated genes by using Genevestigator (performed on February 2021) to obtain a hierarchical clustering by comparing the genes modulated in response to treatment with eDNA against all available data from the database of *S. lycopersicum*. In the group of genes downregulated after treatment with eDNA, almost all downregulated genes are also downregulated in two clusters of genes (Supplementary Figure 3, CL1 and CL2) which are involved in the processes of columella, pericarp, placenta, collenchyma, and parenchyma development. In contrast, several genes downregulated after treatment with eDNA are also downregulated by *Pseudomonas syringae* pv. *tomato* (Supplementary Figure 3, CL3). With regards to genes upregulated after treatment with eDNA, the Genevestigator analysis returns the presence of three clusters (Supplementary Figure 4, CL1, CL2, and CL3) which show upregulation in *S. lycopersicum* processes involved in epidermic, parenchyma, vascular tissues development (Supplementary Figure 4, CL4), and seed development (Supplementary Figure 4, CL5).

### The Response of Tomato Leaves to Tomato eDNA Is the Modulation of Many Genes That Respond to Biotic Stress

Having assessed the involvement of ion homeostasis and tissue development, two important aspects involved in plant responses to pathogens and herbivores, we focused our attention on the analysis of genes expressed in *S. lycopersicum* responses to biotic stress. The hierarchical clustering of eDNA downregulated genes against the *S. lycopersicum* database of Genevestigator shows a high expression potential for genes involved in responses to *P. syringae* pv. *tomato* (Supplementary Figure 5, CL1), nematodes (Supplementary Figure 5, CL2), other pathogens (Supplementary Figure 5, CL3), and responses to pathogen elicitors (Supplementary Figure 5, CL4). The same situation was observed for genes upregulated after treatment with eDNA, with a high expression potential for genes involved in pathogen interactions (Supplementary Figure 6, CL1 and CL3), nematodes (Supplementary Figure 6, CL2), and pathogen elicitors (Supplementary Figure 6, CL4).

### Validation of RNA-Seq Gene Expression by qPCR Reveals a Quantitative Modulation of Biotic Stress-Related Genes Upon Treatment With eDNA

The gene expression obtained by RNA-Seq analysis was validated by qPCR. We selected groups of downregulated and upregulated genes related to different categories related to genes coding for proteins involved in channel activity (Vm variations) and to calcium, potassium, and ROS (CLSM analyses) as well as plant responses to biotic stress.

**FIGURE 6:**
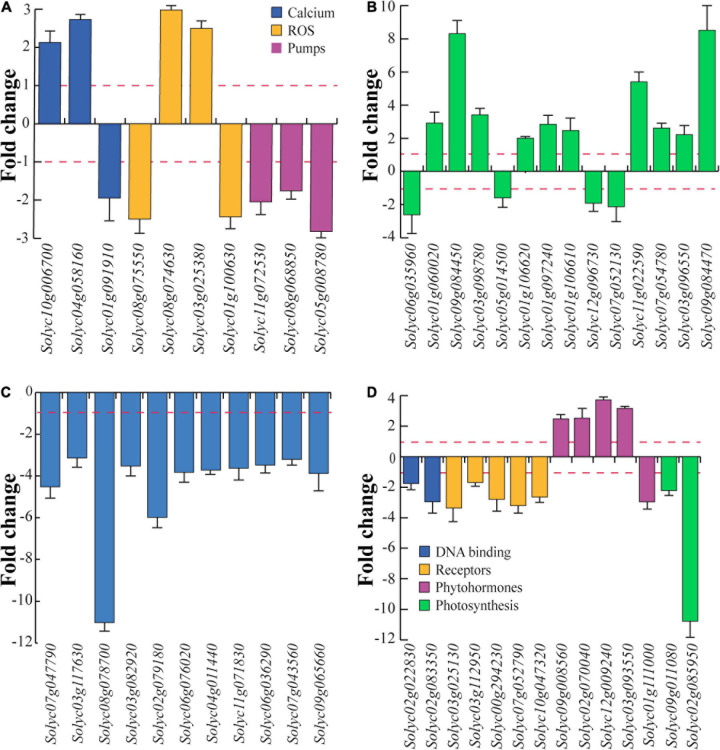
Differential gene expression of tomato selected genes in response to eDNA. The data are expressed as fold change in relation to controls (intact DNA). To emphasize the visualization of data, fold change values below 1 were plotted as –1/value, in order to obtain negative fold change values (indicating downregulation). **(A)** Gene expression of calcium-, ROS-, and proton pump-related genes. **(B)** Genes involved in plant defense. **(C)** Gene expression of heat shock proteins and heat shock factors. **(D)** Gene expression of DNA binding-, receptor like-, phytohormone-, and photosynthesis-related genes. In all figures, the red dotted lines represent the control (intact DNA) level; metric bars indicate standard deviation (*N* = 3).

The first group of genes is related to calcium, ROS, and proton pumps (Figure 6A). A calcium-binding EF hand family protein (*Solyc10g006700*) and calmodulin (*Solyc04g058160*) were upregulated, whereas the calcium-binding phospholipase D (*Solyc01g091910*) was downregulated by treatment with eDNA. Oxidative stress-related genes included ubiquinol oxidase (*Solyc08g075550*) and catalase (*Solyc01g100630*) that were downregulated by treatment with eDNA, whereas the chloroplastic polyphenol oxidase F (*Solyc08g074630*) and a peroxidase (*Solyc03g025380*) were upregulated. With regards to proton pump-related genes, V-type proton ATPase subunit a (*Solyc11g072530*) and two proton pump interactor 1 (*Solyc08g068850* and *Solyc05g008780*) were downregulated (Figure 6A).

Several genes involved in plant defense were regulated by treatment with eDNA (Figure 6B). Downregulation was found for 4-coumarate-CoA ligase-like protein (*Solyc06g035960*), a multidrug resistance protein ABC transporter family (*Solyc05g014500*), polygalacturonase (*Solyc12g096730*), and a sesquiterpene synthase (*Solyc07g052130*). On the other hand, β-1,3-glucanase (*Solyc01g060020*), chymotrypsin inhibitor-2 (*Solyc09g084450*), Kunitz-type protease inhibitor (*Solyc03g098780*), pathogenesis-related protein 1a (*Solyc01g106620*), pathogenesis-related protein P2 (*Solyc01g097240*), pathogenesis-related protein-1 (*Solyc01g106610*), a trypsin inhibitor-like protein precursor (*Solyc11g022590*), a wound-induced protein (*Solyc07g054780*), a wound/stress protein lipoxygenase, LH2 PLAT domain-containing protein (*Solyc03g096550*), and a wound-induced proteinase inhibitor 1 (*Solyc09g084470*) were all upregulated. In particular, a strong upregulation was found for chymotrypsin inhibitor-2, a trypsin inhibitor-like protein precursor and a wound/stress protein lipoxygenase (Figure 6B).

Among the genes regulated in response to eDNA, several Hsps and chaperones were downregulated with particular reference to the mitochondrial heat shock protein 22 (*Solyc08g078700*) and heat shock transcription factor 1 (*Solyc02g079180*) (Figure 6C).

A large number of receptor-like kinases (RLK) was downregulated in response to application of eDNA, including several serine/threonine-protein kinases, leucine-rich repeat (LRR), tyrosine protein kinases as well as toll-interleukin receptors (Supplementary Table 2). Downregulation was confirmed for DNA binding DNA primase/helicase (*Solyc02g022830*) and DNA-directed RNA polymerase (*Solyc02g083350*) as well as for receptor-like serine/threonine-protein kinase (*Solyc03g025130*), serine/threonine-protein kinase (*Solyc03g112950*), TIR-NBS-LRR resistance protein Toll-Interleukin receptor (*Solyc00g294230*), TIR-NBS-LRR disease resistance-like protein (*Solyc07g052790*), and CC-NBS-LRR, resistance protein (*Solyc10g047320*) (Figure 6D). Among phytohormones, ethylene was involved in the upregulation of ACC oxidase (*Solyc09g008560*), ethylene-responsive nuclear protein (*Solyc02g070040*), ethylene-responsive transcription factor 4 (*Solyc12g009240*), and ethylene-responsive TF1 pathogenesis-related transcriptional factor (*Solyc03g093550*), whereas an auxin-induced SAUR-like protein (*Solyc01g111000*) was downregulated in response to application of eDNA (Figure 6D). Finally, a strong downregulation was found for chloroplastic RuBisCO small subunit 3B (*Solyc02g085950*) followed by a twofold downregulation of RuBisCO activase 1 (*Solyc09g011080*) (Figure 6D).

## Discussion

In this work, we provide evidence that the application of extracellular fragmented self-eDNA (eDNA) to tomato leaves induces a typical response to biotic stress, supporting the stimulating hypothesis that some plant responses to pathogens and herbivores might be triggered by the degradation of the plant DNA. Evidence was supported by evaluating both early (Vm variations, calcium and potassium channel activity, and ROS generation) and late events (gene expression).

### eDNA Induces Early Tomato Events Which Are Typical of the Biotic Stress Response

In plants, early events occur within seconds to minutes upon biotic/abiotic stress perception (Maffei et al., 2007). One of the early events in plant interactions with the surrounding environment is the variation in the plasma transmembrane potential (Vm) (Zebelo and Maffei, 2015). The application of eDNA caused a consistent increase of Ca^2+^_cyt_ in treated tomato plants, which is a typical response of plants to both pathogens and herbivores (Bricchi et al., 2012; Singh and Pandey, 2020), including tomato (Zebelo et al., 2012). As a consequence, the Vm was depolarized and a possible calcium-dependent Vm depolarization was followed by the activation of potassium channels, as it is typical in plant–insect and plant–pathogen interactions (Amborabe et al., 2008; Bricchi et al., 2013). The increase of both Ca^2+^ and K^+^ cellular concentrations are associated with the observed Vm depolarization. Moreover, we found that the tomato response to eDNA was a stronger fluorescence of the ligand-gated K^+^ channels, with respect to voltage-gated channels. In plants, K^+^ conductance by ligand-gated channels is restricted in the presence of millimolar concentrations of Ca^2+^ (Leng et al., 2002) and this might explain the reason for the higher activity. This ligand-gated fluorescence could also explain the reason why Vm depolarization is not fully recovered after washing eDNA treated leaves with a fresh buffer solution. We hypothesize that eDNA might bind or interfere with the binding activity of K^+^ channels.

The generation of ROS is another early event in plant–pathogen (Camejo et al., 2019), plant–herbivore interactions (Zebelo and Maffei, 2015), systemic signaling (Fichman and Mittler, 2020), and plant immune response (Kuzniak and Kopczewski, 2020). ROS act as important signal transduction molecules and may act downstream or upstream of several signal transduction pathways (Foyer and Noctor, 2005). Moreover, increases in the production, accumulation, and signaling of ROS are one of the main causes of programmed cell death (Farooq et al., 2019). We observed that the tomato response to eDNA was a ROS production which was localized mainly in the chloroplasts. Besides being involved in biotic stress-induced Ca^2+^ signals (Nomura et al., 2012), during pathogen and herbivore attack, chloroplasts are important sources of ROS (Maffei et al., 2006; Camejo et al., 2016), and the generation of ROS may interfere with several plant cell functions, including photosynthetic and other metabolic processes (Sierla et al., 2012). Interestingly, the strong chloroplastic-localized ROS production was associated to a strong downregulation of the RuBisCO (*Solyc02g085950*) gene expression. Furthermore, the ROS biotic-induced production is transmitted to chloroplasts via calcium ions that play an important role in regulating nuclear gene expression, making ROS important modulators of the plant immune response (Zabala et al., 2015). Therefore, we can conclude from our results that the tomato response to eDNA involves most of the early events triggered by biotic stress, including the alteration of the membrane potential due to the increased levels of Ca^2+^_cyt_, which causes the opening of voltage-gated K^+^ channels and the regulation of ligand-gated K^+^ channels; these early events are followed by a ROS production which are mainly localized in the plant cell chloroplasts.

### The Tomato Early Responses to eDNA Treatment Are Followed by Modulation of Biotic Stress-Related Genes

Transcriptomic analyses of leaves treated with eDNA reveal a significant modulation of tomato genes. A consistent downregulation was found for most of the processes involved in the plant–biotic response, including the generation of ROS and jasmonate involvement, but other important processes were also affected like the cell wall biosynthetic process and the sucrose transport (see Supplementary Figure 1). In contrast, ion homeostasis and responses to pathogens were upregulated (Supplementary Figure 2). A deeper search in the Genevestigator database confirmed the correlation between up- and down-regulation of genes in response to eDNA and plants’ responses to pathogens, pathogens’ elicitors and nematodes (Supplementary Figures 3–6).

The validation of RNA-Seq analysis allowed us to focus on specific biotic stress-related genes. The positive correlation between the CLSM calcium signature (Figure 2) and the upregulation of both calcium-binding EF-hand family protein member (*Solyc10g006700*) that is homologous to a *S. tuberosum* calcium-binding protein which is involved in resistance to *Phytophthora infestans* (Muktar et al., 2015) and calmodulin (*Solyc04g058160*) (Figure 6A) is interesting. EF-hand motifs in the hydrophilic C-terminal domain of tomato have been correlated to Ca^2+^_cyt_ regulation of ROS production (Amicucci et al., 1999), whereas calmodulin, a calcium-binding protein with a helix-loop-helix (EF-hand) motif and one of the key mediators in plant immune responses (Cheval et al., 2013), is required for a successful defense response to pathogens (Zheng et al., 2018) and is upregulated by *Botrytis cinerea* infection (Yu and Du, 2018) a plant pathogen that causes cell disruption and death (Camejo et al., 2016; Oren-Young et al., 2021). On the other hand, the downregulation of the calcium-binding phospholipase D (*Solyc01g091910*), a protein that hydrolyses different membrane phospholipids and that is implicated in plant–pathogen interactions (Zhao, 2015), has also been shown to interfere with ethylene signaling regulation (Dek et al., 2018). The ROS signals work downstream from Ca^2+^ (Farooq et al., 2019) and the Ca^2+^_cyt_ increase was associated with the upregulation of chloroplastic polyphenol oxidase F (*Solyc08g074630*) and a peroxidase (*Solyc03g025380*). Polyphenol oxidase upregulation is involved in tomato resistance to herbivory (Lin et al., 2021) and pathogens (Zhang and Sun, 2021), whereas peroxidase activity was associated to tomato resistance to early blight disease (Alizadeh-Moghaddam et al., 2020). On the opposite, ubiquinol oxidase (*Solyc08g075550*) and catalase (*Solyc01g100630*) were downregulated in response to eDNA treatment. In plants, the alternative oxidase catalyzes the oxidation of ubiquinol and reduces oxygen avoiding the proton translocation by bypassing some steps in the respiratory pathway (Finnegan et al., 2004). Since the ubiquinol oxidase activity reduces the ATP production, we suppose that the downregulation of this gene might be a plant cell strategy to cope with the altered homeostasis due to eDNA action and provide more ATP for proton pump activity. On the other hand, the catalase downregulation is directly correlated to the increased ROS production, being catalase one of the major ROS scavengers in plants (Fones and Preston, 2012).

The plant cell Vm is maintained by the activity of the proton pump (Falhof et al., 2016). We recently showed that during plant–herbivore interactions, the Vm depolarization is sustained by the strongly reduced effects of insect’s oral secretions on the interaction between H^+^-ATPase and 14-3-3 proteins, suggesting that one of the leading players in biotic stress-dependent Vm depolarization is the inhibition of the proton pump (Camoni et al., 2018). The V-type proton ATPase (*Solyc11g072530*) interacts with 14-3-3 proteins (Klychnikov et al., 2007) while the tomato proton pump interactors (*Solyc08g068850* and *Solyc05g008780*) are regulated by abiotic stress (Garcia et al., 2011). The downregulation of these genes in response to eDNA treatment was positively correlated with the Vm depolarization caused by application of eDNA (Figures 1, 6A).

### The Tomato Response to eDNA Involves the Modulation of the Expression of Genes Involved in Plant Late Responses to Biotic Stress

The signal transduction pathway that uses calcium and ROS as second messengers eventually leads to the regulation of defense response genes. Besides the typical biotic stress-responsive genes like pathogenesis-related proteins (PRPs) (*Solyc01g106620*, *Solyc01g097240*, and *Solyc01g106610*), including β-1,3-glucanase (*Solyc01g060020*), a strong upregulation was also found for a series of proteinase inhibitors (PIs) (*Solyc09g084450*, *Solyc03g098780*, *Solyc11g022590*, and *Solyc09g084470*). PIs play a vital role in defenses against pests and pathogens, especially against herbivores and, in tomato, PI genes have been recently found to mediate the response of tomato to biotic stress by balancing hormone signals (Fan et al., 2020). Another response to tomato to eDNA was the downregulation of 4-coumarate-CoA ligase-like protein (*Solyc06g035960*) which encodes for an enzyme that thioesterifies coumaric acid to coenzyme A (CoA) to form coumaroyl CoA, the precursor of a vast diversity of phenylpropanoids (Alberstein et al., 2012). The function of this gene has also been correlated to its ability to impair membrane functions such as ion transport (Kienow et al., 2008). Another intriguing plant response to eDNA is the downregulation of a polygalacturonase (*Solyc12g096730*) a gene that encodes an enzyme that catalyzes the hydrolysis and disassembly of pectin in plant cell walls (Caffall and Mohnen, 2009). Suppression of the gene can repress the pectin depolymerization and change the postharvest pathogen susceptibility (Ke et al., 2018).

### Treating Tomato With eDNA Downregulates Genes Coding for Calcium-Dependent Protein Kinases, Heat Shock Transcription Factors, and Heat Shock Proteins

Many molecular chaperones are stress proteins and many of them were originally identified as heat shock (HS) proteins (Hsp), with particular reference to abiotic stress (Wang et al., 2004). Heat shock transcription factors (Hsfs) family members exert their anti-stress effects by regulating a series of H molecular chaperones, and other functional protein genes (Kovtun et al., 2000) and Hsp expression result from the binding of an Hsf to the HS element (HSE) in the promoter region of Hsp genes (von Koskull-Doring et al., 2007). Treatment with eDNA prompted the downregulation of the heat shock transcription factor 1 (*Solyc02g079180*) and heat stress transcription factor A3 (*Solyc09g065660*), which along with the downregulation of calcium-dependent protein kinases (CPK1, *Solyc03g031670*, Supplementary Table 2), was associated to the downregulation of several small and large Hsps. In plants, a correlation between calcium binding activity and Hsf has been demonstrated. For instance, some CPKs phosphorylate Hsfs which promote the transcriptional activation of plant defense genes (Kanchiswamy et al., 2010b) and the impairment of CPK downregulates the expression of several Hsps (Kanchiswamy et al., 2010a). In tomato, Hsfs regulate a wide range of metabolic pathways and have been identified as major players in physiological development in response to stress (Paupiere et al., 2020). Although Hsps play a major role in abiotic stress responses (e.g., to heat) as molecular chaperones, Hsps are involved in protein folding and in avoiding the irreversible aggregation of denatured proteins (Sun et al., 2002). In tomato, the activation of Hsps prevents lipid peroxidation, the generation of excessive reactive radicals and increases the secretion of plant antioxidant enzymes (Khan et al., 2020). Therefore the downregulation of all Hsps in response to eDNA treatment might be associated to the reduced scavenging activity and the increased production of ROS. Downregulation of Hsps, with particular reference to small Hsps, like the strongly downregulated heat shock protein 22 (*Solyc08g078700*), has been observed upon herbivory (Bricchi et al., 2012), confirming their involvement also in biotic stress.

### The Tomato Response to eDNA Is the Modulation of Receptor-Like Protein Kinases and Ethylene-Responsive Factors

Our transcriptomic analysis reveals that plants respond to eDNA treatment by modulating several other genes involved in plant responses to biotic stress. In plants, pathogen-associated molecular patterns (PAMPs) and DAMPs are mainly recognized via receptor-like kinases (RLKs) (Duran-Flores and Heil, 2018). RLKs were largely downregulated (Supplementary Table 2). RLKs play a role both in abiotic stress (e.g., cold, salt, and drought tolerance) (Ye et al., 2017) and resistance to infection by several pathogens (Bundó and Coca, 2016). In tomato, RLKs are involved in biotic stress and knockdown of an RLK resulted in increased sensitivity to fungi and reduced resistance against the pathogen *B. cinerea* (Xu et al., 2020). Interestingly, a significant downregulation was found for TIR-NBS-LRR Toll-Interleukin receptor (*Solyc00g294230*) (Supplementary Table 2). Emerging evidence suggests that TLR-mediated signal transduction pathways lead to the movement of calcium deposits through calcium channel activity (Liu et al., 2008; Zhang et al., 2013).

Ethylene is a crucial phytohormone involved in plant responses to biotic stress as well as in tomato fruit development (Liu et al., 2021). In tomato, ethylene is induced by the pathogenic fungus *Oidium neolycopersici* (Kissoudis et al., 2016) and by *P. syringae* pv. *tomato* (Moran-Diez et al., 2020), the latter being a pathogen that causes a rapid and localized programmed cell death (PCD) (Moyano et al., 2020). Ethylene biosynthesis involves the conversion of 1-aminocyclopropane-1-carboxylic acid (ACC) to ethylene, a reaction catalyzed by ACC oxidase (Boller et al., 1979). The plant response to eDNA was the upregulation of ACC oxidase (*Solyc09g008560*) and the same modulation was found for several ethylene-responsive transcription factors (*Solyc02g070040*, *Solyc12g009240*, and *Solyc03g093550*) (Figure 6D and Supplementary Table 2). Ethylene-responsive factors (ERFs) belong to a subfamily of the AP2/ERF superfamily which is involved in tomato response to the pathogens *P. syringae* pv. *tomato* (He et al., 2001) and *Stemphylium lycopersici* (Yang et al., 2021), among others.

## Conclusion

Plant reactions to biotic stress encompass signal transduction cascades, receptors, and biochemical pathways involved in responding to pathogens and herbivores. Recent reports suggest that the application of fragmented pathogen DNA may have an impact when applied in crop protection strategies to cope with pathogens (Serrano-Jamaica et al., 2021). Here we show that not only pathogen fragmented DNA but also self-eDNA induces plant responses typical of biotic responses to pathogens and herbivores. The early and late responses induced by treatment of tomato leaves with tomato eDNA imply the “recognition of small-sized nucleotide molecules” as suggested by several authors (Duran-Flores and Heil, 2018; Heil and Vega-Munoz, 2019; Monticolo et al., 2020) and the involvement of CPKs, RLKs, ERFs, ion homeostasis (calcium, and potassium involvement) and ROS production demonstrated in this work are strongly consistent with this proposition. Moreover, the ROS production induced by eDNA may trigger further DNA degradation and PCD events, which would reinforce the plant response to eDNA. Our results support the intriguing hypothesis that some of the plant reactions to pathogens and herbivores might be due to the plant cell DNA degradation, particularly when associated to the plant cell disruption. Passive cell disruption by chewing herbivores and pathogen-triggered necrotic cell death might be a realistic scenario for the release of self-DNA fragments as DAMPs. Pathogen-inflicted cell lysis including the degradation of host DNA by pathogen-derived DNAses has been bought forward already in 1993 (Gerhold et al., 1993) and since then supported by diverse follow-up studies (Gerhold et al., 1993; Isaac et al., 2009; Hadwiger and Chang, 2015). Moreover, necrotrophic pathogens use DNAse to digest their host’s DNA as a source of nutrients (and thereby liberate a DAMP that triggers a defense response), and recent studies suggest that pathogens or herbivores can use DNAses as an effector that removes a DAMP and thereby allows them to escape from the detection by the plant immune system (Huang et al., 2019; Park et al., 2019). In summary, there is some interesting evidence for a role of eDNA also in non-controlled (i.e., non-apoptotic) cell death due to (necrotrophic) pathogens or a (chewing) herbivore and fragmented DNA would then become an important and powerful elicitor able to trigger early and late responses to biotic stress.

## Data Availability Statement

The datasets presented in this study can be found in online repositories. The names of the repository/repositories and accession number(s) can be found below: European Nucleotide Archive (ENA) under the accessions ERX5409868, ERX5409869, ERX5409870, and ERX5409871.

## Author Contributions

MM conceived and designed the study and drafted the manuscript. FB, MM, MI, and MG performed the experiments. FB revised it critically. All authors approved the final version of the article.

## Conflict of Interest

The authors declare that the research was conducted in the absence of any commercial or financial relationships that could be construed as a potential conflict of interest.

## Publisher’s Note

All claims expressed in this article are solely those of the authors and do not necessarily represent those of their affiliated organizations, or those of the publisher, the editors and the reviewers. Any product that may be evaluated in this article, or claim that may be made by its manufacturer, is not guaranteed or endorsed by the publisher.

## Footnotes

1
http://www.qiagen.com/

2 https://www.bioinformatics.babraham.ac.uk/projects/fastqc/

## Supplementary Material

Supplementary Figure 1 Gene ontology (GO) of the biological processes of downregulated genes by fold enrichment.

Supplementary Figure 2 Gene ontology (GO) of the biological processes of upregulated genes by fold enrichment.

Supplementary Figure 3 Hierarchical clustering by comparing the genes downregulated by treatment of plants with eDNA against the Genevestigator database of *S. lycopersicum*.

Supplementary Figure 4 Hierarchical clustering by comparing the genes upregulated in response to eDNA against the Genevestigator database of *S. lycopersicum*.

Supplementary Figure 5 Hierarchical clustering of the expression potential of genes downregulated in response to eDNA against the *S. lycopersicum* database of Genevestigator.

Supplementary Figure 6 Hierarchical clustering of the expression potential of genes upregulated in response to eDNA against the *S. lycopersicum* database of Genevestigator.

Supplementary Table 1 Primers used in this work.

Supplementary Table 2 Tomato genes downregulated and upregulated by application of eDNA.
