# Supplementary figures and images for: Extracellular Fragmented Self-DNA Is Involved in Plant Responses to Biotic Stress
Source: Front Plant Sci. 2021 Jul 26;12:686121. doi: 10.3389/fpls.2021.686121 (PMC8350447; doi:10.3389/fpls.2021.686121)

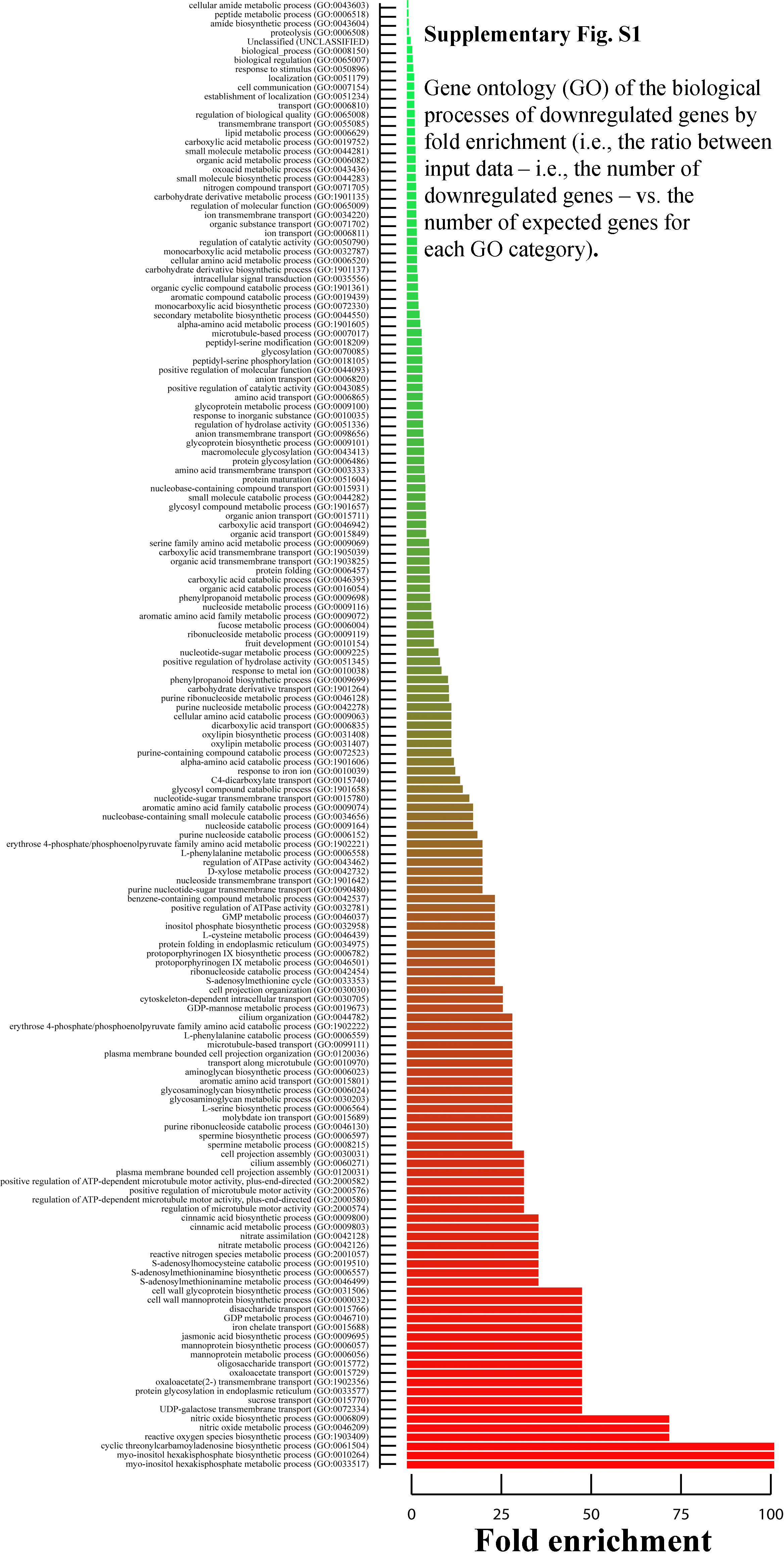

Supplement: Supplementary Figure 1 — Gene ontology (GO) of the biological processes of downregulated genes by fold enrichment. [file Image_1.TIF]

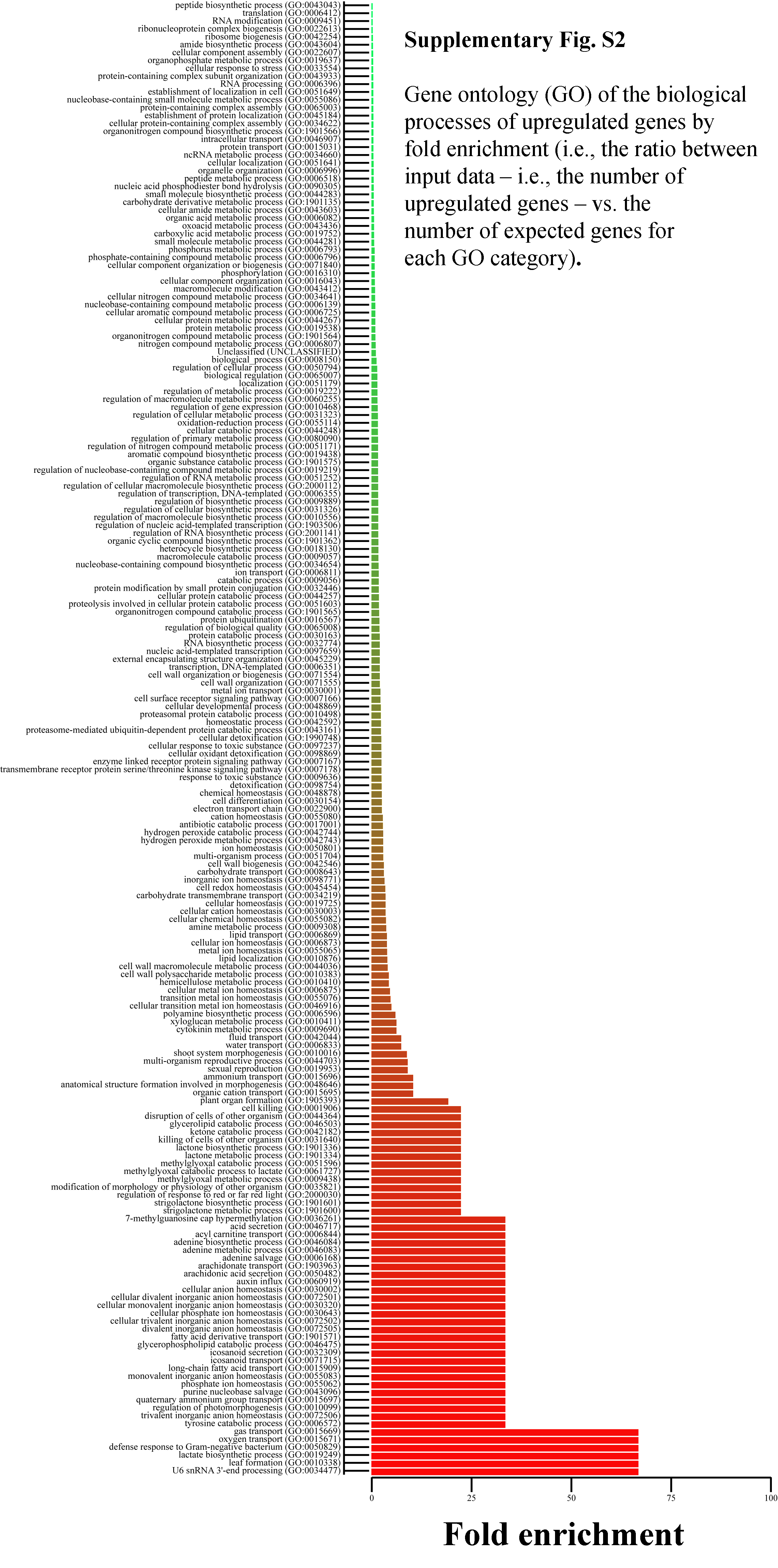

Supplement: Supplementary Figure 2 — Gene ontology (GO) of the biological processes of upregulated genes by fold enrichment. [file Image_2.TIF]

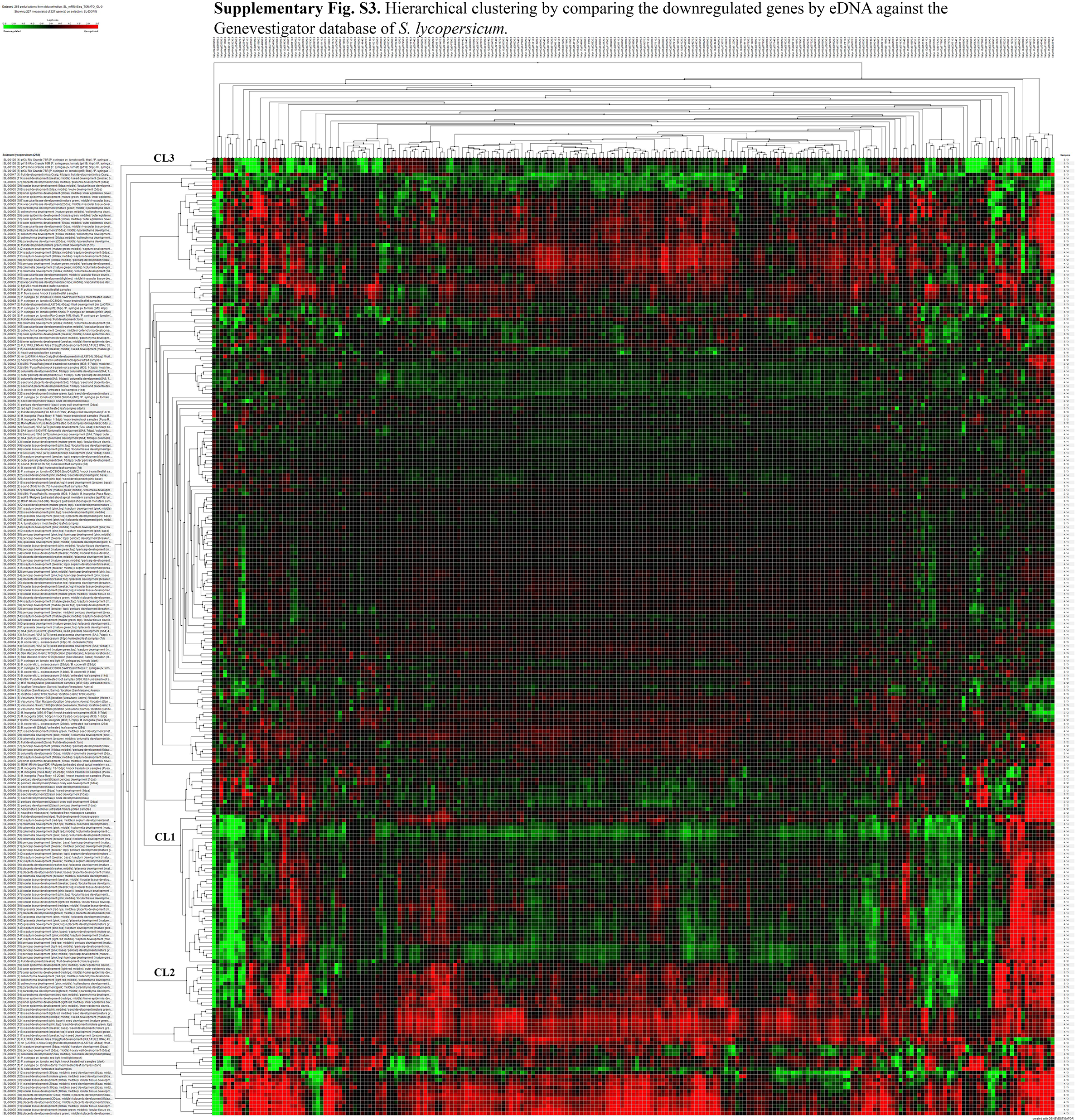

Supplement: Supplementary Figure 3 — Hierarchical clustering by comparing the genes downregulated by treatment of plants with eDNA against the Genevestigator database of S. lycopersicum. [file Image_3.TIF]

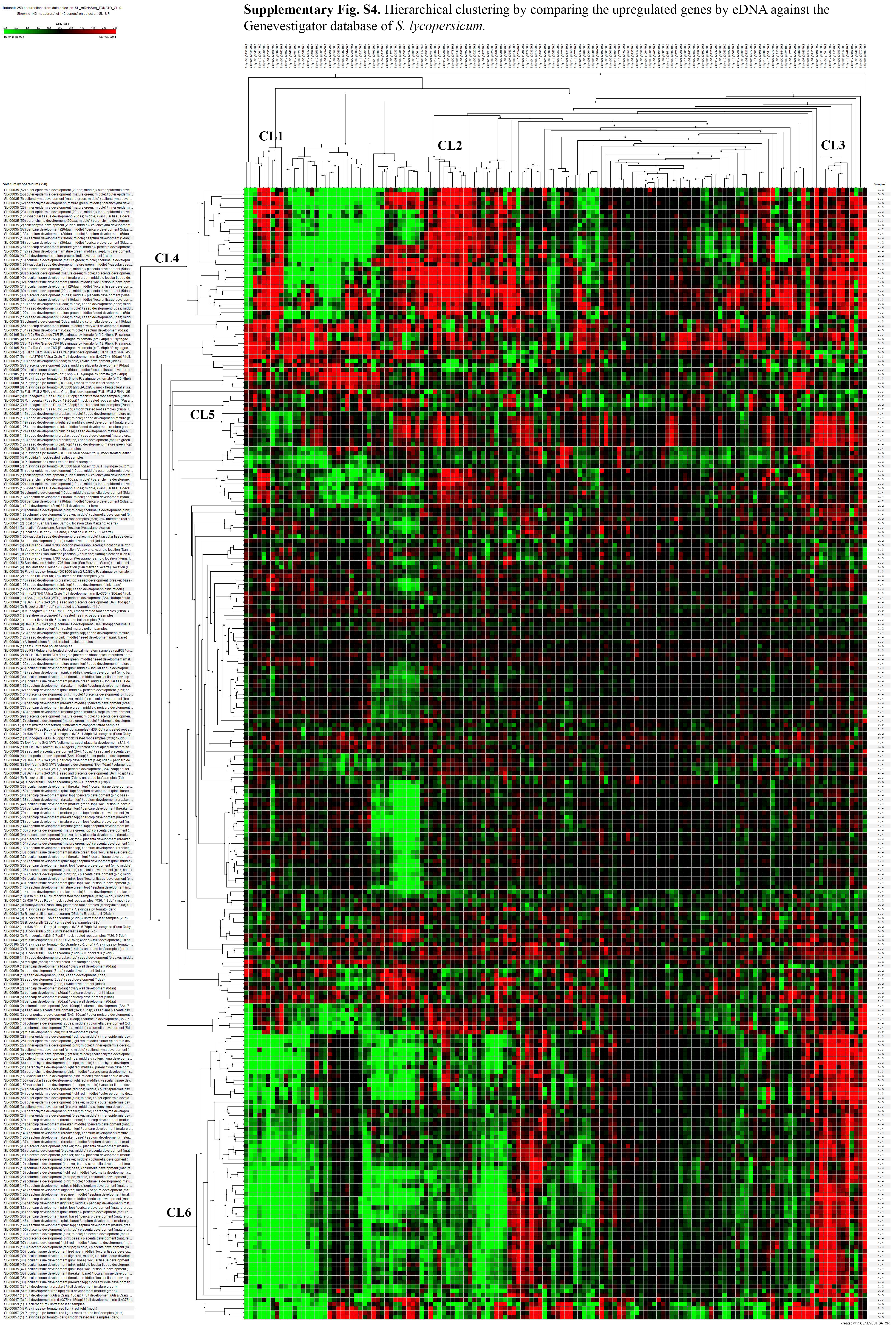

Supplement: Supplementary Figure 4 — Hierarchical clustering by comparing the genes upregulated in response to eDNA against the Genevestigator database of S. lycopersicum. [file Image_4.TIF]

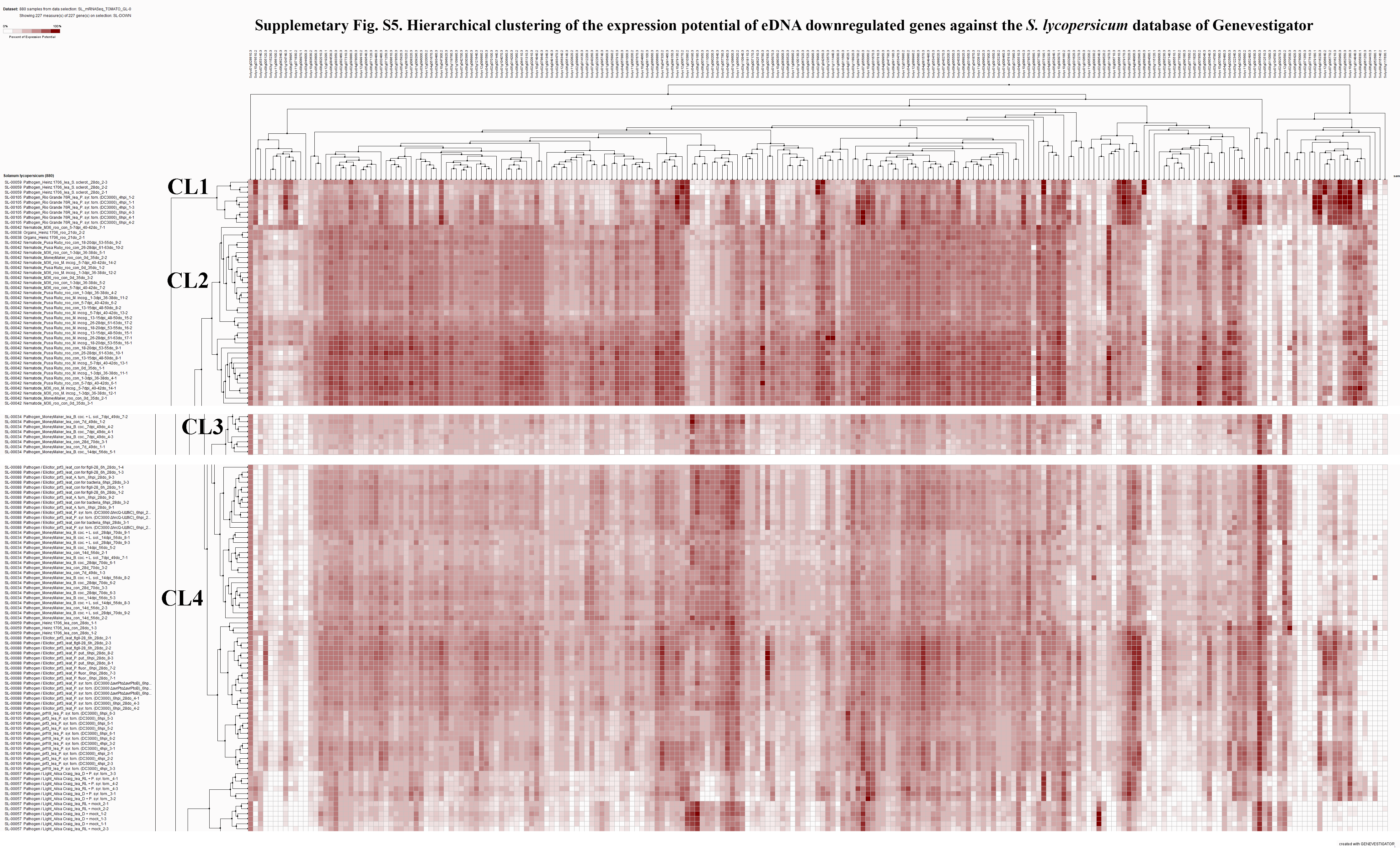

Supplement: Supplementary Figure 5 — Hierarchical clustering of the expression potential of genes downregulated in response to eDNA against the S. lycopersicum database of Genevestigator. [file Image_5.TIF]

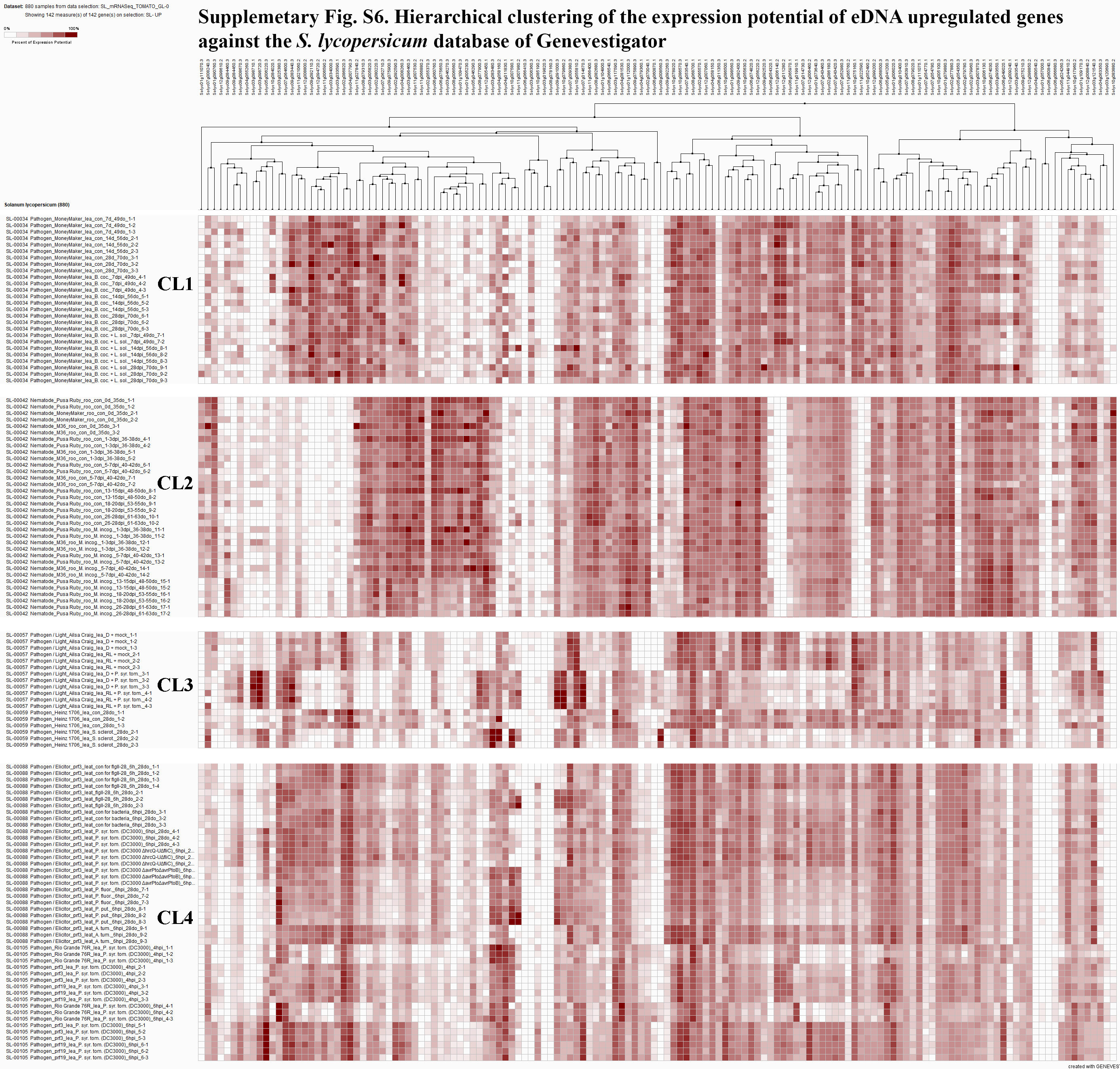

Supplement: Supplementary Figure 6 — Hierarchical clustering of the expression potential of genes upregulated in response to eDNA against the S. lycopersicum database of Genevestigator. [file Image_6.TIF]
