# Supplementary material for: Extracellular Fragmented Self-DNA Is Involved in Plant Responses to Biotic Stress
Source: Front Plant Sci. 2021 Jul 26;12:686121. doi: 10.3389/fpls.2021.686121 (PMC8350447; doi:10.3389/fpls.2021.686121)
Supplement: Supplementary Table 1 — Primers used in this work. [file Table_1.DOCX]

**Supplementary Table S1.** Primers used in this work

| **Category** | **Gene ID** | **Forward** | **Reverse** |
| --- | --- | --- | --- |
| **ROS** |  |  |  |
| Catalase | Solyc01g100630 | GAATGTGTTGTTCATGCCCG | CCGCCATCCAGACTCTATTC |
| Peroxidase | Solyc03g025380 | CACCATCTTCCAGTCTTGCA | GTGTCGTTGTAGATCCGGTT |
| Polyphenol oxidase F, chloroplastic, PPO | Solyc08g074630 | CTCCTATGACGAAACTCCGC | CCAAGAGGGTCAAAAGGGTC |
| Ubiquinol oxidase, | Solyc08g075550 | TACTAGAAACGGTTGCAGCC | ATCCGTTCGTTCTCTGCTTC |
| **Proton pump** |  |  |  |
| Proton pump interactor 1 | Solyc05g008780 | GGCTCGCAAACCAATTCAAA | CGCTTTCTCAGTTCCTCCTC |
| Proton pump interactor 1 | Solyc08g068850 | CCTGATGAAAAGCCACTGGT | CGCTAACCTCTTGCTGTTCT |
| V-type proton ATPase subunit a | Solyc11g072530 | CTTTCCTTTTCGCGGTGATG | CATCTCCATAAAGCTGCCCA |
| **Calcium** |  |  |  |
| Phospholipase D | Solyc01g091910 | AAACTTTGAGGAACGCTGGT | TAGGGTCATCACTGCTCACA |
| DNA primase/helicase | Solyc02g022830 | CGGGAGCATGCTAGAAAACT | CCCTCTTCAAACTCCTGAGC |
| DNA-directed RNA polymerase | Solyc02g083350 | CGGGTAAGTGACGAAGACTG | CATCATCACAGAAGGCCGAA |
| Calmodulin | Solyc04g058160 | GAACGAAAGCCATATCCAAAGG | TAAGATTGGAGCTTGCACGG |
| Calcium-binding EF hand family protein | Solyc10g006700 | GGATAGGGATAAAGGGGCCA | TCCATCACCATCAAAATCCCC |
| **DNA binding** |  |  |  |
| DNA primase/helicase | Solyc02g022830 | CGGGAGCATGCTAGAAAACT | CCCTCTTCAAACTCCTGAGC |
| DNA-directed RNA polymerase | Solyc02g083350 | CGGGTAAGTGACGAAGACTG | CATCATCACAGAAGGCCGAA |
| **Defense-related genes** |  |  |  |
| beta-1,3-glucanase | Solyc01g060020 | GGATCGAACAGGAGGAGGAT | AGGCTTTCTCGGACTACCTT |
| Pathogenesis-related protein P2 | Solyc01g097240 | CTACCTGGGATGCTGACAAG | TGGTCACCCTCAAGCATCTA |
| Pathogenesis-related protein-1 | Solyc01g106610 | ACGATGGTCTAGCAGCCTAT | CATCGTCCCACATCTTCACA |
| Pathogenesis-related protein 1a | Solyc01g106620 | TACGCCAATCAAAGAGCTGG | TCAACCCACATCTTCACAGC |
| Wound/stress protein Lipoxygenase, LH2 PLAT domain-containing protein | Solyc03g096550 | AGAACAACTGTGGCCGTAAG | TGAGCGAAATTCTGAGCCTG |
| Kunitz-type protease inhibitor | Solyc03g098780 | TGAAACTCTAGGCGGGGTAT | CCCTTACGGCAAAATGGACA |
| Multidrug resistance protein ABC transporter family | Solyc05g014500 | GTGTGGGTCAAAGGCAGATT | ACATTGTCCGTCTCTGTATCC |
| 4-coumarate-CoA ligase-like protein | Solyc06g035960 | ATTACTGAGCTCGCTTGGTG | GGATCTGGTGAATGGTAGGC |
| Sesquiterpene synthase | Solyc07g052130 | TGGATTTGGTGATGAGGCAG | TTCACATTCATTGGCTCCCA |
| Wound induced protein | Solyc07g054780 | GAAGAGCGTGGATAGTAGCAG | TGTTGTGCCAAAGACCTCAA |
| Chymotrypsin inhibitor-2 | Solyc09g084450 | TGAAACTCTCATGGCACGAA | GCAAAGGAAAACAATTCTGGCC |
| Wound-induced proteinase inhibitor 1 | Solyc09g084470 | ACTCTCATGGCACGAAAAGA | GTTCTGGCCACATTTGTTTTCC |
| trypsin inhibitor-like protein precursor | Solyc11g022590 | GGTAGTGGAGGTGGTCTTGT | ACGTCCAGTGTTAAGTTCCTG |
| Polygalacturonase | Solyc12g096730 | GTGAAAGCCCCAACACTGAT | CACACGCGACATTTTGGATC |
| **Phytohormones** |  |  |  |
| Auxin-induced SAUR-like protein | Solyc01g111000 | TCACCATACCCTGCAGAGAG | ATCCTGCTTTGCTGCTCTAC |
| Ethylene-responsive nuclear protein | Solyc02g070040 | TCCACCTTCCCCTGTATGTT | CTTCAGAAGCGCGACTAACA |
| Ethylene responsive transcription factor 1a Pathogenesis-related transcriptional factor and ERF, DNA-binding | Solyc03g093550 | AACGGAAGCCATCTCTGAAC | CCTCTGTCTAACTCCCCTGT |
| 1-aminocyclopropane-1-carboxylate oxidase | Solyc09g008560 | ATGGTGGATGTTGGAGGAAG | AGCTTGAGAAAGTCCCTCCA |
| Ethylene-responsive transcription factor 4 | Solyc12g009240 | GGGTTCTTATGATACGCCGG | GAGTCATCGTCCTTCCGTTC |
| **Recceptor** |  |  |  |
| Receptor-like serine/threonine-protein kinase | Solyc03g025130 | AGAAAACCCCGACACGTATC | CGAAATGGAGGGCTCAGATT |
| Serine/threonine-protein kinase | Solyc03g112950 | GTATGGCATGGGATTCACGT | TGTGCTTGCTTTAAAGTGCG |
| TIR-NBS-LRR disease resistance-like protein | Solyc07g052790 | AAGCATTTGGATCTACGCCA | CTGCAATGTCCAAGAGAGGG |
| Cc-nbs-lrr, resistance protein | Solyc10g047320 | ACTCTCTCCACCATAGCTCC | AACGACGCAGCAGACTTATT |
| **Heat shock proteins and Chaperones** |  |  |  |
| Heat shock transcription factor 1 | Solyc02g079180 | CTTCGTCTGTCAGCTCAACA | CCTCAACCACTGTCCTTTCC |
| Heat shock protein Heat shock protein 70 | Solyc03g082920 | GAAACAGCCTGGAGACGTAC | TGTGGCGGTTTCAATCTTCT |
| heat shock protein | Solyc03g117630 | ACAAGACTGCTGGTGTGAAG | TGTACCTCTCCGCTTCTTGA |
| hsc70.3 er21 ethylene-responsive heat shock protein cognate 70 | Solyc04g011440 | TCCAAGGAACACCACGATTC | ATCTCTGGTCCTGGCTCTTT |
| hsp90 heat shock protein 90 | Solyc06g036290 | AAAGAACTTGAAGCTGGGCA | TCTGACCCTCCTTCATCCTG |
| hsc1 heat shock protein 70 kD Heat shock cognate 70 kDa protein 1 | Solyc06g076020 | GACAAGAGCACCGTTCATGA | AGAGTTCCTTGCCGTTGAAG |
| NEF Heat shock protein 4 Heat shock protein 70 | Solyc07g043560 | AGTATGCTCTTGGATTGGCG | CCTTTCCTTTCCGCTACTCC |
| GRP94 Chaperone protein htpG  Heat shock protein Hsp90 | Solyc07g047790 | CTGATGAAGGCCCAAACTGT | AGGCCTCTTCATCATCTGGA |
| Heat shock protein 22 Mitochondrial small heat shock protein LEMTSHP | Solyc08g078700 | GGAGTTGGAGCAAGAAGAGG | ACTCTTTCTCGCCTTCTCCT |
| SolycHsfA7 Heat stress transcription factor A3 | Solyc09g065660 | CGGAGAGGCTAACGAATTCC | ATCCGTGTTCCCAAATTGCT |
| Hsp40 Chaperone protein dnaj Heat shock protein DnaJ | Solyc11g071830 | GACAAAGAGAGAGGAGGGGT | CCTTGCCACTACACTTCGAG |
| **Photosynthesis** |  |  |  |
| Ribulose bisphosphate carboxylase small chain 3B, chloroplastic, RuBisCO small subunit 3B, | Solyc02g085950 | TGATTTGTCCGACGAGCAAT | TGCCATCGTAGTATCCTGGT |
| Ribulose-1 5-bisphosphate carboxylase/oxygenase activase 1 | Solyc09g011080 | ACTCCCCGGTATGTACAACA | TCTTCTCTAGTTGGTGCCCA |
| **Housekeeping genes** |  |  |  |
| ACT1 | TC194780a | GAAATAGCATAAGATGGCAGACG | ATACCCACCATCACACCAGTAT |
| EF1 | X14449 | GGAACTTGAGAAGGAGCCTAAG | CAACACCAACAGCAACAGTCT |
| TUB | DQ205342 | AACCTCCATTCAGGAGATGTTT | TCTGCTGTAGCATCCTGGTATT |
| UBI | TC193502a | GGACGGACGTACTCTAGCTGAT | AGCTTTCGACCTCAAGGGTA |
